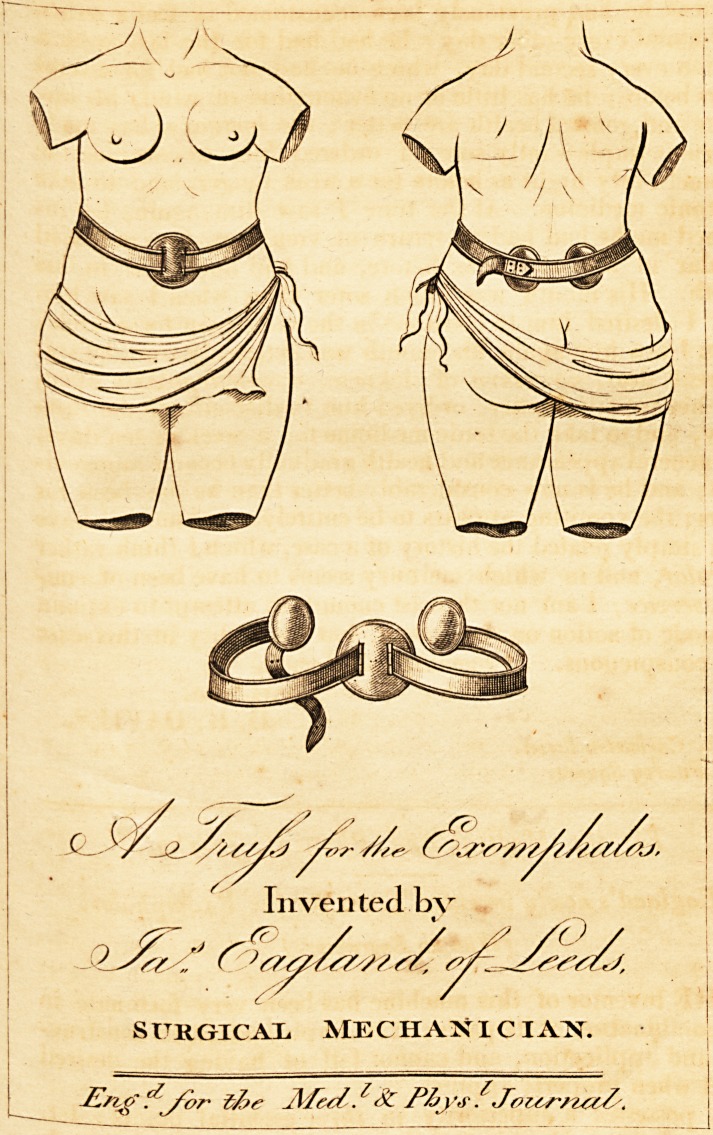# Eagland's Newly Invented Truss for the Exomphalos

**Published:** 1811-04

**Authors:** 


*For the Medical and Physical Journal.*

### *Eagland's newly invented Truss for the Exomphalos.*

*(With an Engraving.)*

THE inventor of this machine has been very fortunate in the combination of its parts; it is simple both in its construction and application, and cannot fail of having the desired effect when properly applied.

It possesses a superiority in *three* essential points; 1st. The certainty of keeping on the part for which it is designed. 2d. Of being perfectly easy to the wearer. 3d. The springs being japanned, are perfectly secure from corrosion by perspiration.

The *principle* of this machine is equally applicable in cases of

*A Truſs for the Exomphalos.*

Invented by

*Ja.s Eagland, of Leeds,*

SURGICAL MECHANICIAN.

*Eng.d for the Med.l & Phys.l Journal.*

of ventral, or even in compound cases of hernia; as it may be adapted to any part of the abdomen.

Mr. Hey has given a description of its construction in the second edition of his *Practical Observations in Surgery*, p. 577. And in the Preface to the above valuable work, the author has spoken in high terms of Mr. Eagland's machines for supporting the *spine*, and the extremities of the body, in various deformities.

## Figures and Tables

**Figure f1:**